# Intracardiac Ewing-Like Sarcoma: A Diagnostic and Therapeutic Challenge

**DOI:** 10.7759/cureus.101698

**Published:** 2026-01-16

**Authors:** Victor Oyervides-Ortiz, Leonel Gomez-Llanos, Antonio Garza-Cruz, Ivett Miranda-Maldonado, Victor Oyervides-Juarez

**Affiliations:** 1 Medicine, Centro Universitario Contra el Cáncer, University Hospital “Dr. José Eleuterio González" Autonomous University of Nuevo León, Monterrey, MEX; 2 Internal Medicine, Centro Universitario Contra el Cáncer, University Hospital “Dr. José Eleuterio González" Autonomous University of Nuevo León, Monterrey, MEX; 3 Pathology, University Hospital “Dr. José Eleuterio González" Autonomous University of Nuevo León, Monterrey, MEX; 4 Oncology, Centro Universitario Contra el Cáncer, University Hospital “Dr. José Eleuterio González" Autonomous University of Nuevo León, Monterrey, MEX

**Keywords:** cardiac sarcoma, ewing-like sarcoma, ewing sarcoma family of tumors(esft), intracardiac tumor, small round blue cell tumors

## Abstract

Ewing-like sarcomas are rare malignancies that typically arise in bone or soft tissue, with intracardiac presentations being exceptionally uncommon and associated with diagnostic uncertainty and high mortality due to anatomical constraints and therapeutic challenges. We report the case of a 22-year-old male who presented with progressive dyspnea and was found to have a large biatrial mass originating from the interatrial septum, resulting in atrial obstruction, pericardial effusion, and reduced systolic function. Subtotal surgical resection revealed a malignant small round cell tumor with an immunohistochemical profile positive for CD99 and NKX2.2, consistent with an Ewing-like sarcoma, although molecular confirmation was not available. Postoperative treatment with systemic chemotherapy was complicated by severe neutropenia, ventilator-associated pneumonia, and superimposed cardiogenic compromise, culminating in death on hospital day 35. Intracardiac Ewing-like sarcomas represent a diagnostically challenging and highly aggressive entity with a grave prognosis due to limited surgical resectability, hemodynamic compromise, and heightened susceptibility to treatment-related complications. This case highlights the narrow therapeutic window in patients with cardiac involvement and underscores the importance of multidisciplinary evaluation and careful administration of intensive therapy.

## Introduction

Ewing sarcoma (ES) is a malignant neoplasm of bone and soft tissue thought to arise from mesenchymal stem cells [1-3]. It belongs to the group of undifferentiated small round cell sarcomas and is molecularly defined by gene fusions between EWSR1 or FUS and ETS family transcription factor genes, most frequently FLI1 (>90%) or ERG, as described by the World Health Organization [4,5]. Although rare, ES is the second most common malignant primary bone tumor in children and the third most common in the overall population [6-8]. Extraskeletal Ewing sarcoma (EES) is approximately 10 times less common than bone ES and shows a predilection for the paravertebral soft tissues, lower extremities, head and neck region, and pelvis [9,10].

Clinically, EES most commonly affects children and young adults and presents as rapidly enlarging soft-tissue or osseous masses, often associated with pain or compressive symptoms, whereas visceral involvement is extremely rare [11]. Radiologically, these tumors typically appear as large, ill-defined, heterogeneous masses with areas of necrosis; however, no imaging feature is pathognomonic, and definitive diagnosis requires pathological confirmation [12]. Histologically, these tumors consist of uniform small round blue cells with low mitotic activity and variable pseudorosette formation, occasionally showing a fibrillary matrix and Homer-Wright rosettes. Immunohistochemical markers demonstrate strong membranous CD99 expression and nuclear positivity for NKX2.2, with molecular confirmation of EWSR1 rearrangement necessary to distinguish them from other Ewing-like sarcomas harboring CIC or BCOR alterations [13-15].

Current management follows multimodal ES protocols, incorporating intensive multi-agent chemotherapy combined with surgical resection and/or radiotherapy. Staging of EES uses MRI for local evaluation (before biopsy), and chest CT plus whole-body imaging for metastases, with FDG-PET/CT preferred for detecting bone disease and treatment response. Definitive classification requires image-guided biopsy with histopathology and molecular confirmation of an EWSR1 rearrangement. EES generally has a better prognosis than skeletal ES, particularly in localized disease, with higher five-year overall survival. Poor prognostic factors include older age, pelvic location, large tumor size, adverse laboratory markers, poor histologic response to neoadjuvant chemotherapy, and metastatic disease, whereas extremity location, surgical resection, and good chemotherapy response are favorable; recurrence is associated with an almost uniformly fatal outcome [11]. We report an exceptionally rare intracardiac Ewing-like tumor, highlighting diagnostic challenges and therapeutic considerations in this unusual presentation.

## Case presentation

A 22-year-old male from Monterrey, Nuevo León, Mexico, with no significant personal or family medical history and no history of exposure to biomass or industrial smoke, presented with a six-month history of progressive dyspnea on exertion. Over the weeks preceding admission, his symptoms had worsened to dyspnea with minimal activity, accompanied by chest tightness and orthopnea. He denied alcohol consumption, tobacco use, or illicit drug use.

On arrival at the emergency department, the patient was tachypneic and hypoxemic. Vital signs were as follows: blood pressure: 155/87 mmHg, heart rate 126 beats per minute, temperature: 36.7 °C, and oxygen saturation: 89% on room air. Chest radiography demonstrated cardiomegaly with an increased cardiothoracic ratio. The admission electrocardiogram revealed atrial fibrillation with a right bundle branch block and Q waves, without clear electrocardiographic evidence of acute ischemia.

**Figure 1:**
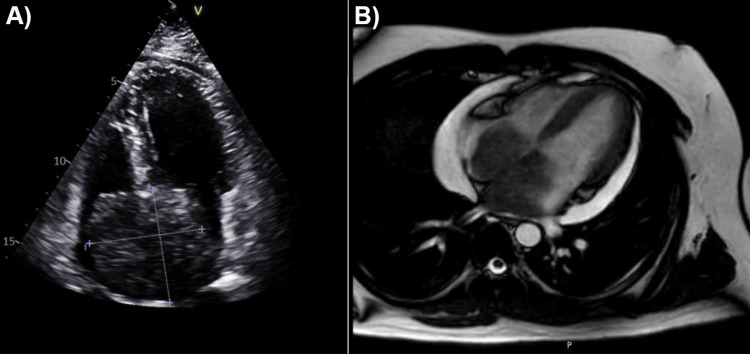
Cardiac imaging findings A) Transthoracic echocardiography: apical four-chamber view, demonstrating a large hyperechogenic intracardiac mass arising from the interatrial septum and extending into both atria. B) Cardiac magnetic resonance imaging: large biatrial mass with a moderate circumferential pericardial effusion

Transthoracic echocardiography revealed a large hyperechoic mass measuring 6.0 × 6.1 cm arising from the interatrial septum, extending into both atrial chambers, and occupying approximately 90% of their volume. Subsequent cardiac MRI further characterized the lesion and demonstrated a moderate pericardial effusion with a maximal separation of 22.3 mm. Although the patient presented with dyspnea and hypoxemia, there was no echocardiographic or hemodynamic evidence of cardiac tamponade; the symptoms were attributed to mechanical obstruction by the tumor. Cardiac MRI also showed mildly reduced left ventricular systolic function, with an estimated ejection fraction of 48% (Figure 1).

Ten days after admission, the patient underwent subtotal resection of the cardiac mass via median sternotomy on cardiopulmonary bypass with biatrial access. Given the tumor’s extensive infiltration of the interatrial septum, resection was limited to debulking to relieve obstruction and obtain diagnostic tissue 

**Figure 2:**
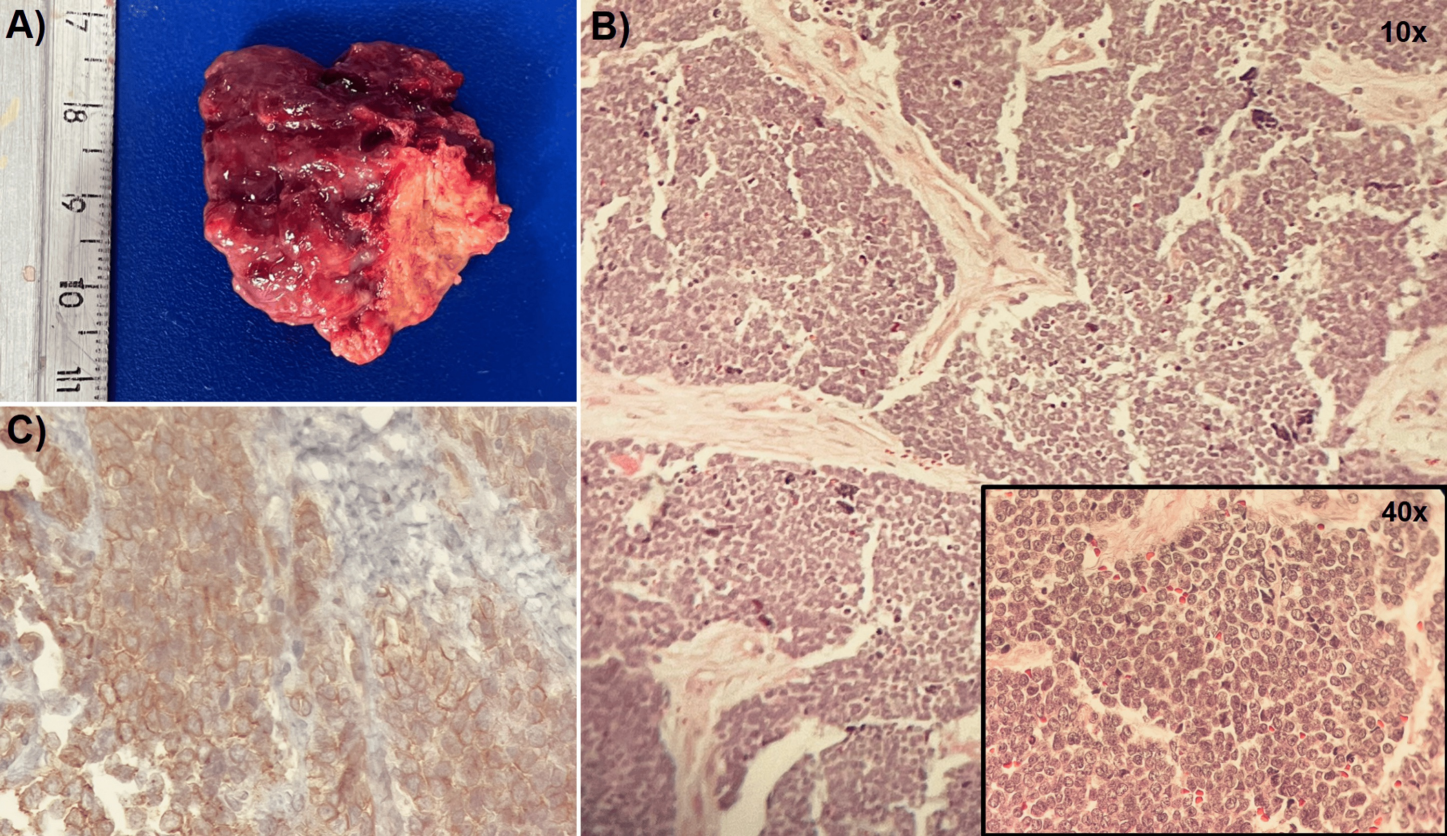
Pathologic findings of the intracardiac tumor A) Gross macroscopic view of the tumor following subtotal surgical resection. B) Hematoxylin and eosin-stained sections (original magnification 10x and 40x) showing a uniform population of small round blue cells. C) Immunohistochemical staining for CD99 demonstrating strong diffuse membranous positivity. Other immunohistochemical stains were performed as part of the diagnostic workup; however, representative images were not available for inclusion

Histopathological examination revealed a malignant small round cell neoplasm composed of uniform small round blue cells. Immunohistochemical analysis demonstrated strong membranous CD99 expression and nuclear positivity for NKX2.2, while tumor cells were negative for CD3, CD45 (LCA), CD20, terminal deoxynucleotidyl transferase (TdT), cytokeratins, synaptophysin, and placental alkaline phosphatase (PLAP). Representative NKX2.2 immunohistochemistry images were not available for inclusion. In the absence of molecular cytogenetic testing, these morphologic and immunophenotypic features were most consistent with an Ewing sarcoma-like tumor (Figure 2).

On hospital day 23, the patient began systemic chemotherapy with vincristine, doxorubicin, and cyclophosphamide (VDC), administered according to standard Ewing sarcoma protocols, with doses calculated based on body surface area. Despite ongoing hematologic monitoring, the white blood cell count continued to decline, and on hospital day 31, a complete blood count revealed profound neutropenia, with an absolute neutrophil count of 0.01 × 10⁹/L, accompanied by a fever of 38.5 °C. The patient received supportive care, including granulocyte-macrophage colony-stimulating factor (GM-CSF), blood product transfusions, and prophylactic antibiotics.

On hospital day 34, the patient developed ventilator-associated pneumonia, with multiplex polymerase chain reaction testing indicating infection with IMP-producing Pseudomonas aeruginosa, for which empiric therapy with piperacillin-tazobactam was initiated. He subsequently met criteria for septic shock, with a Sequential Organ Failure Assessment (SOFA) score of 10 and a serum lactate level of 8.9 mmol/L. Despite continuous vasopressor infusion, the patient remained hypotensive. Laboratory evaluation revealed markedly elevated N-terminal pro-B-type natriuretic peptide levels (>35,000 ng/L) and a mild elevation of high-sensitivity troponin I (0.07 ng/mL, slightly above the 99th-percentile upper reference limit), suggesting superimposed cardiogenic shock in the setting of severe underlying cardiac disease, recent cardiac surgery, and sepsis.

On hospital day 35, the patient's condition worsened, and despite maximal vasopressor support, broad-spectrum antimicrobial therapy, and advanced critical care interventions, he died from circulatory collapse and multiorgan failure secondary to septic shock complicated by tumor-related cardiac dysfunction.

## Discussion

Ewing sarcoma is a rare malignancy, with an estimated annual incidence of approximately one to three cases per million population. It occurs predominantly in children, adolescents, and young adults, with a peak incidence between 10 and 20 years of age, and is uncommon after the third decade of life. A strong racial and ethnic predilection has been consistently observed, with Ewing sarcoma occurring far more frequently in individuals of European ancestry, while remaining rare in Asian and African populations. While most tumors arise in bone, 10-20% are extraosseous, most often involving the soft tissues of the trunk and extremities, with rarer visceral and organ-based sites also described [8,16].

Current evidence supports a mesenchymal stem/progenitor cell origin for Ewing-spectrum tumors, in which EWS-ETS fusions block normal mesenchymal differentiation and transcriptionally redirect tumor cells toward varying degrees of neural-like phenotype. Within this framework, classic Ewing sarcoma represents the undifferentiated end of the spectrum, whereas peripheral primitive neuroectodermal tumors correspond to a more differentiated neural phenotype. These differences are therefore phenotypic rather than lineage-defining, arising from oncogene-driven transcriptional modulation rather than distinct cells of origin. Oncogenic EWS-ETS fusions may drive tumorigenesis in diverse anatomic sites, with local microenvironmental factors influencing the degree of phenotypic differentiation [1-3]. This framework explains the occurrence of rare extraosseous and anatomically critical presentations, such as the intracardiac involvement observed in this case.

The differential diagnosis of a malignant small round cell tumor includes lymphoma, rhabdomyosarcoma, synovial sarcoma, desmoplastic small round cell tumor, and neuroendocrine neoplasms [14]. These entities were excluded based on the absence of hematolymphoid, myogenic, epithelial, and neuroendocrine marker expression. In contrast, the characteristic morphology combined with strong membranous CD99 and nuclear NKX2.2 positivity supported classification within the Ewing sarcoma family, consistent with an extraosseous Ewing-like sarcoma. While CD99 lacks specificity when used alone, NKX2.2 has emerged as a relatively sensitive and specific marker for Ewing-spectrum tumors, particularly in extraosseous sites. As a direct transcriptional target of the EWS-FLI1 fusion, NKX2.2 is consistently upregulated in EWS-rearranged tumors, providing indirect molecular evidence of EWS-ETS fusion-driven transcriptional activity when cytogenetic confirmation is unavailable, as in this case [13,15].

Although only approximately 25% of patients present with overt metastases, Ewing sarcoma is considered a systemic disease, necessitating multimodal therapy. When treated with contemporary multimodal regimens, extraskeletal Ewing sarcoma demonstrates overall survival outcomes comparable to skeletal disease. Large retrospective series using standard vincristine, doxorubicin, and cyclophosphamide-based regimens alternating with ifosfamide and etoposide (VDC-IE) report five-year overall survival rates of approximately 65-70% for localized extraskeletal tumors, similar to skeletal Ewing sarcoma. However, extraskeletal presentations are consistently associated with higher rates of local recurrence and lower local-recurrence-free survival, particularly in visceral or anatomically constrained sites, reflecting challenges in achieving optimal local control rather than intrinsic chemoresistance [8,17,18].

The extensive biatrial involvement observed in this patient resulted in severe functional impairment, as evidenced by atrial obstruction, arrhythmia, pericardial effusion, and reduced left ventricular systolic function. Even after subtotal surgical resection, the residual tumor burden and recent cardiac surgery likely left minimal physiologic reserve. These factors may have contributed to the rapid progression to mixed shock once systemic infection developed, highlighting the vulnerability of patients with intracardiac malignancies to critical illness.

Despite initiation of guideline-consistent multi-agent chemotherapy, the patient developed severe treatment-related myelosuppression, culminating in ventilator-associated pneumonia and septic shock. While intensive chemotherapy remains the cornerstone of therapy for Ewing-spectrum tumors, this case illustrates the narrow margin between therapeutic benefit and life-threatening toxicity in patients with compromised cardiac function and postoperative status. The fatal outcome reflects the convergence of several adverse prognostic factors, including primary cardiac location, extensive intracardiac involvement, inability to achieve complete surgical resection, and development of severe infectious complications. Although early multimodal treatment was pursued, the aggressive disease biology combined with an anatomically critical tumor site ultimately limited therapeutic success.

## Conclusions

Intracardiac Ewing sarcoma-like tumors represent an exceptionally rare and highly lethal manifestation within the Ewing sarcoma spectrum. This report highlights the diagnostic challenges posed by extraosseous Ewing-family tumors in anatomically critical locations, particularly when cytogenetic confirmation is unavailable, and underscores the value of integrated morphologic and immunophenotypic assessment, including CD99 and NKX2.2 expression, as surrogate markers of fusion-driven disease. The patient’s rapid clinical deterioration illustrates how tumor location can profoundly limit physiologic reserve and therapeutic tolerance, despite early surgical intervention and guideline-consistent multimodal therapy. These observations emphasize the importance of early multidisciplinary evaluation, careful risk-benefit assessment of intensive treatment strategies, and transparent acknowledgment of diagnostic uncertainty. Reporting such cases provides valuable insights into the clinical behavior and management limitations of Ewing-family tumors in settings where standard treatment paradigms may be insufficient.
